# Correction to: “Should We Adopt the Case Report Format to Report Challenges in Complicated Evidence Synthesis? A Proposal and Illustration of a Case Report of a Complex Search Strategy for Humanitarian Interventions” and “A New Process Model of Study Identification Specific to the Identification of Randomised Studies for Systematic Reviews of Medical Interventions”

**DOI:** 10.1002/cesm.70090

**Published:** 2026-06-24

Cooper C, Premji Z, Yavuz C, Engelbert M. Should we adopt the case report format to report challenges in complicated evidence synthesis? A proposal and illustration of a case report of a complex search strategy for humanitarian interventions. Cochrane Ev Synth. 2025;3:e70021. doi:10.1002/cesm.70021

Cooper, C., Premji, Z., Worsley, C., Tomlinson, E., Dawson, S. and Prentice, E. (2025), A New Process Model of Study Identification Specific to the Identification of Randomised Studies for Systematic Reviews of Medical Interventions. Cochrane Evidence Synthesis and Methods, 3: e70026. https://doi.org/10.1002/cesm.70026.

In the originally published version of the above articles, the Conflicts of Interest (COI) statement was inadvertently omitted.

The following COI statement has now been added to the articles:

## Conflicts of Interest

The authors declare no conflicts of interest.

This correction has been made to complete the disclosure record. The content and conclusions of the article remain unchanged.

Wiley apologise for this error.

